# Integrative bioinformatics and artificial intelligence analyses of transcriptomics data identified genes associated with major depressive disorders including *NRG1*

**DOI:** 10.1016/j.ynstr.2023.100555

**Published:** 2023-07-07

**Authors:** Amal Bouzid, Abdulrahman Almidani, Maria Zubrikhina, Altyngul Kamzanova, Burcu Yener Ilce, Manzura Zholdassova, Ayesha M. Yusuf, Poorna Manasa Bhamidimarri, Hamid A. AlHaj, Almira Kustubayeva, Alexander Bernstein, Evgeny Burnaev, Maxim Sharaev, Rifat Hamoudi

**Affiliations:** a Research Institute for Medical and Health Sciences, University of Sharjah, Sharjah, United Arab Emirates; b Applied AI Center, Skolkovo Institute of Science and Technology, Moscow, Russian Federation; c The Center for Cognitive Neuroscience, Al Farabi Kazakh National University, Kazakhstan; d Faculty of Medicine, University of Sharjah, Sharjah, United Arab Emirates; e Division of Surgery and Interventional Science, University College London, London, United Kingdom; f ASPIRE Precision Medicine Research Institute Abu Dhabi, University of Sharjah, Sharjah, United Arab Emirates

**Keywords:** Depression biomarkers, Major depressive disorder, Bioinformatics, Machine learning, Gene expression, Brain regions

## Abstract

Major depressive disorder (MDD) is a common mental disorder and is amongst the most prevalent psychiatric disorders. MDD remains challenging to diagnose and predict its onset due to its heterogeneous phenotype and complex etiology. Hence, early detection using diagnostic biomarkers is critical for rapid intervention. In this study, a mixture of AI and bioinformatics were used to mine transcriptomic data from publicly available datasets including 170 MDD patients and 121 healthy controls. Bioinformatics analysis using gene set enrichment analysis (GSEA) and machine learning (ML) algorithms were applied. The GSEA revealed that differentially expressed genes in MDD patients are mainly enriched in pathways related to immune response, inflammatory response, neurodegeneration pathways and cerebellar atrophy pathways. Feature selection methods and ML provided predicted models based on MDD-altered genes with ≥75% of accuracy. The integrative analysis between the bioinformatics and ML approaches identified ten key MDD-related biomarkers including *NRG1, CEACAM8, CLEC12B, DEFA4, HP, LCN2, OLFM4, SERPING1, TCN1* and *THBS1*. Among them, *NRG1*, active in synaptic plasticity and neurotransmission, was the most robust and reliable to distinguish between MDD patients and healthy controls amongst independent external datasets consisting of a mixture of populations. Further evaluation using saliva samples from an independent cohort of MDD and healthy individuals confirmed the upregulation of *NRG1* in patients with MDD compared to healthy controls. Functional mapping to the human brain regions showed *NRG1* to have high expression in the main subcortical limbic brain regions implicated in depression. In conclusion, integrative bioinformatics and ML approaches identified putative non-invasive diagnostic MDD-related biomarkers panel for the onset of depression.

## Highlights

• AI-associated computational tools have the potential to detect, and improve the treatment of complex psychiatric disorders.
• Integrative analysis using bioinformatics and ML on transcriptomics data identified promising MDD-related biomarkers.
• *NRG1* was identified as a putative non-invasive liquid biopsy biomarker for the diagnosis of MDD patients.

## 1 Introduction

Major Depressive Disorder (MDD) is among the most prevalent, chronic complex psychiatric disorders nowadays (Baxter et al., 2013; Smith, 2014). Depression affects over 280 million people globally according to the World Health Organization (WHO, 2023), and is the second leading cause of disability worldwide after cancer, while predicted to be the leading cause by 2030 (Malhi and Mann, 2018). In the post-COVID-19 era, mental and behavioral disorders are reported to have become more severe, possibly due to the pandemic effects on healthcare and the economy worldwide (Liang et al., 2022; Poletti et al., 2022).

MDD is a heterogeneous disorder, resulting from a complex interaction of social, psychological, environmental and genetic factors (GOLDBERG, 2011; Alhaj et al., 2008). Depression does not only affect the mental and psychological aspects of the individual's health, but also affects physical health by disturbing the heart, kidney, nervous system, and immune system (Trivedi, 2004). It is characterized by the presence of depressed moods, functional impairments, a loss of interest in activities, fatigue, sleep disturbances, and psychomotor retardation or agitation (Buyukdura et al., 2011; Maj et al., 2020). This negatively affects the patient's productivity, self-perception, and self-esteem, resulting in an impaired quality of life which can lead to suicidal ideation and attempt. The WHO reported that more than 700,000 individuals worldwide die as a result of suicide each year, with depression being a leading cause (2023). Thus, MDD has grown into a major public health problem that needs urgent attention.

Despite extensive research, the pathophysiology of MDD is still poorly understood. Around 40% of MDD patients do not show an adequate response or remission to anti-depressant treatment and ultimately develop treatment resistance which further exacerbates the disease (Baxter et al., 2013). Additionally, the diagnosis of MDD continues to be made on clinical assessment, which can be assisted by psychiatric questionnaire-based tools rather than laboratory-based tests which may be associated with several limitations and heterogeneity of the illness (Maj et al., 2020; Nelson et al., 2018). Indeed, self-administered questionnaire-based approaches are not sufficient in evaluating efficiently the differences across patients' sub-groups and identifying patients' depressive stages. Adding to the fact that the absence of precise diagnostic biomarkers has resulted in a complex MDD diagnosis with other etiologically associated disorders such as bipolar disorder (Mitchell et al., 2009; Hashimoto and Makowski, 2018).

The use of functional neuroimaging techniques including functional magnetic resonance imaging (fMRI) and electroencephalogram (EEG) offer potential means for the assessment of the changes in brain activity associated with MDD as well as the prediction of response to treatment (Alhaj et al., 2011; Dunlop and Mayberg, 2017; Kustubayeva et al., 2022). However, these techniques are yet to be utilized in clinical practice due to multiple factors, including impracticality, expensive costs and limited specificity. Therefore, it is of great importance to screen for sensitive and specific biomarkers to improve MDD diagnostics and treatment.

Studies have shown that MDD affects the functional activity of the brain which can be identified by analyzing data on aberrations in the neuronal network (Alhaj et al., 2007; Wang et al., 2017). Moreover, the heterogeneity of depressive disorders may also be related to neuronal plasticity correlated with different depressive symptomatology (Honer, 1999). Besides, it is suggested that MDD results from systemic changes in the signaling and biochemical pathways involved in the regulation of moods, cognitive functions and disposition (Palazidou, 2012). Therefore, a comprehensive understanding of affected or associated biological pathways involved in MDD is of high importance to reveal the molecular mechanism of MDD and to identify accurate targets for the MDD diagnosis.

To date, machine learning (ML) as an application of AI approaches is successfully applied in medical studies, by training computer models to process and understand complex patterns within big-data which facilitate classifications or predictions of new cases (MacEachern and Forkert, 2021; Joyce et al., 2021). Moreover, using OMICs strategies is amongst the most applied approaches at the forefront of personalized medicine in psychiatry with high efficiency (Joyce et al., 2021; Ressler and Williams, 2021), such as transcriptomics which showed increasing evidence of the potential to detect, and substantially improve the treatment of such complex cognitive disorders (Johnson et al., 2019; Li et al., 2021a). Furthermore, there is a rising interest in using integrative OMICs and neuroimaging data to understand how an altered genetic profile in psychiatric disorders could influence brain structure and function. This can provide a deeper understanding of molecular mechanisms and uncover endophenotypes-neurobiological changes because of genes implicated in depression (Buch and Liston, 2021).

In this study, the key aim is to compare transcriptomics data of MDD patients to healthy controls using bioinformatics and ML approaches and to identify key biomarkers that can aid in the diagnosis and prediction of the onset of patients with depression.

## 2 Materials and methods

### 2.1 Transcriptome MDD datasets

The Gene Expression Omnibus database (GEO) was searched for publicly available MDD and healthy controls (HCs) transcriptomic datasets (https://www.ncbi.nlm.nih.gov/geo/) to choose the appropriate gene expression datasets. The selection criteria included studies for Homo sapiens and MDD patients recruited as per international practice guidelines in psychiatry. Studies with no healthy control samples or with a large batch effect were excluded. The GSE98793 dataset, which met all the inclusion criteria was selected and processed (Leday et al., 2018). The samples were performed in two batches, for which batch information was extracted from phenotypic data. This dataset consists of high-resolution gene expression sets retrieved from whole blood samples of 128 MDD patients and 64 HCs. The Affymetrix Human Genome U133-Plus 2.0 gene expression gene-chip was used. The MDD patients were diagnosed if at least two episodes of depression satisfying DSM-IV or ICD10 criteria were identified using the semi-structured Schedule for Clinical Assessment in Neuropsychiatry (SCAN). The demographic and clinical details of the GSE98793 dataset are listed in Supplementary Table 1.

### 2.2 Raw microarray normalization and adaptive filtering

**Fig. 1:**
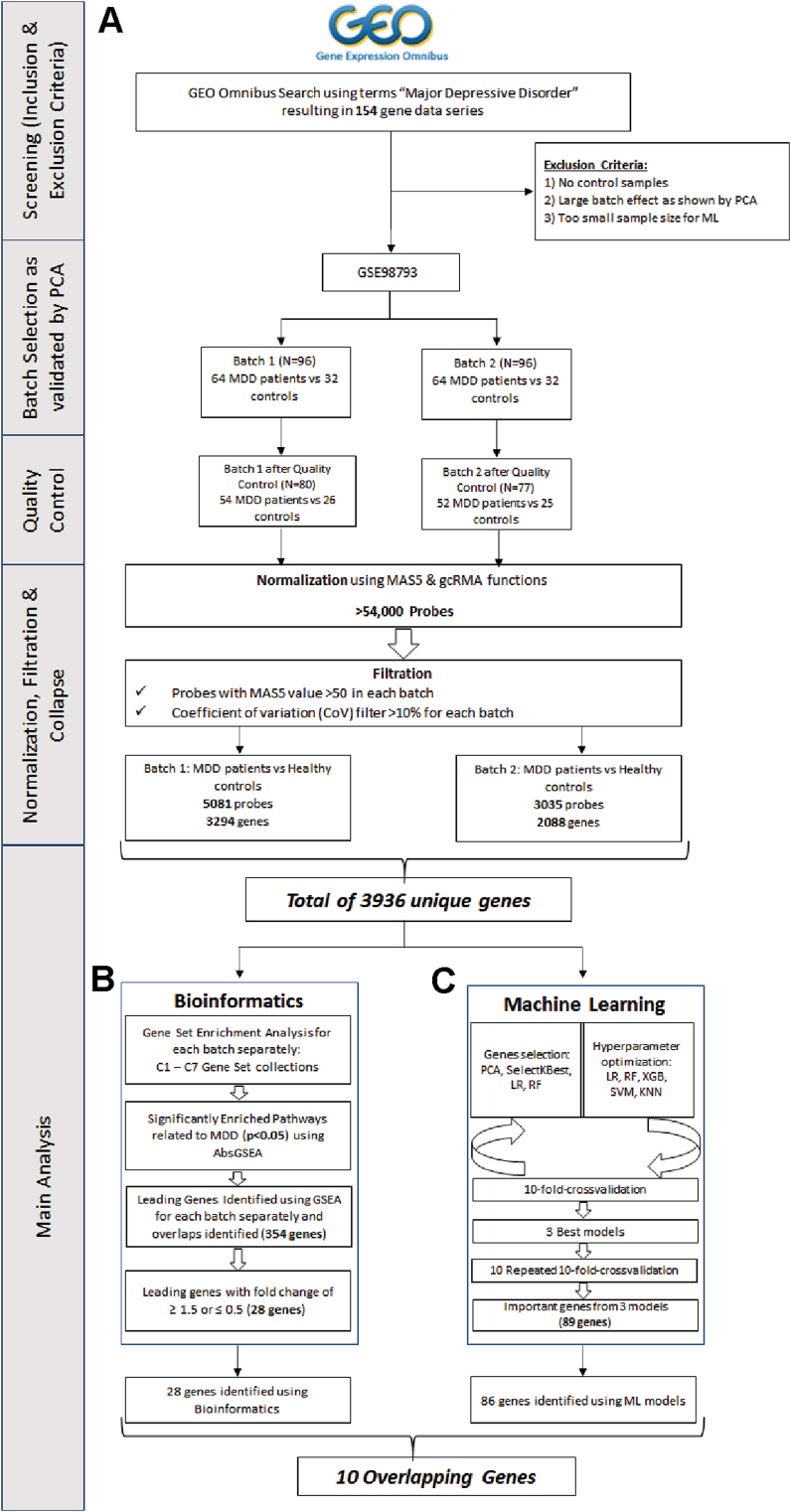
Overview of the bioinformatics and Machine Learning workflow. A) Data processing of MDD and healthy controls transcriptomic data. B) Bioinformatics analysis including GSEA. C) Machine Learning Pipeline.

The Affymetrix microarray covers more than 54,000 probes. The raw CEL files were downloaded and analyzed using an in-house R script pipeline. First, logarithmic transformation and batch effect correction were performed to eliminate the experimental effect on the datasets. Next, to normalize and filter out the background noise, MAS5 and GCRMA packages from the Bioconductor project (https://www.bioconductor.org/) were applied. Non-specific filtering was carried out to extract the common variant probes, while adaptive filtering was applied to identify probes with MAS5 value > 50 and a coefficient of variation >10% in GCRMA. Then, processed probes were intersected across all MDD patients and HCs to identify common variant probes. The filtered common probes between all samples from the two batches of the dataset were combined into a data matrix and mapped to the genes list using Broad Institute software (https://www.gsea-msigdb.org/gsea/index.jsp). For the collapse of gene symbols, the probes with maximum expression were selected as the expression value for the gene. Probes mapping to housekeeping genes or not assigned to any gene were excluded. The workflow of the bioinformatics analysis is shown in Fig. 1A.

### 2.3 Gene set enrichment analysis

The mapped gene expression list was thereafter subjected to the absolute Gene Set Enrichment Analysis (GSEA) to identify the activated and enriched cellular pathways in MDD patients in comparison to HCs Fig. 1B. The absolute GSEA search was carried out on the expression data as previously described (Hamoudi et al., 2010), using around 25,828 annotated cellular pathways obtained across seven well-annotated gene sets obtained from the Human Molecular Signatures Database (MSigDB). The significantly identified pathways were then subjected to the normal GSEA considering the significance cut-off of p-value *p* < 0.05. The significantly activated pathways were further processed to identify the differentially enriched genes between the MDD patients and HCs. As parameters, we used; random permutations = 1000, permutation type = sample labels and enrichment correlation-based weighting = 1. Top leading gene analysis was applied for all significant gene sets to identify the core leading genes for each pathway. Only genes, with fold change (FC) > 1.5 considered as upregulated and FC < 0.5 considered as downregulated, were further explored in the downstream analysis.

### 2.4 Machine learning pipeline

ML analysis was performed on the same mapped gene expression list from the GSE98793 dataset using SkLearn v.1.2.1 library in Python v3.9. To examine various ML models with different feature selection (FS) approaches a common ML pipeline was applied (Sharaev et al., 2018). The ML pipeline selection involves FS and hyperparameter optimization for different ML models including Logistic Regression (LR), Random Forest (RF), XGBoost (XGB), Support Vector Machine Classifier (SVM) and K-Nearest Neighbors (KNN) with 10-fold cross-validation (Fig. 1C). The prediction performance of each classification model was estimated by accuracy, F1 score and ROC-AUC measures. The accuracy is the proportion of correctly predicted subjects among all subjects. Its formula is presented below:

$$Accuracy=\frac{TP+TN}{TP+TN+FP+FN}$$

where TP = True Positive; FP = False Positive; TN = True Negative; FN = False Negative

The F1 score is the harmonic mean of precision and recall. The precision is the fraction of truly predicted subjects related to a certain class among all subjects for which a classification model was assigned to this class. The recall is the fraction of truly predicted subjects related to a certain class among all subjects related to this class. We used the F1-macro score, where we averaged all per-class F1 scores.

$$Precision=\frac{TP}{TP+FP}$$

$$Recall=\frac{TP}{TP+FN}$$

$$F1=2\frac{precision*recall}{precision+recall}$$

ROC-AUC is the area under the error curve, which evaluates the quality of the model without being tied to a specific threshold. For building the ROC curve, TPR against the FPR is plotted, and then the area under the resulting curve was calculated.

$$TPR=\frac{TP}{TP+FN}$$

$$FPR=\frac{FP}{FP+TN}$$

### 2.5 Features selection using ML

Transcriptomic data consists of a large number of genes (features) that are much more than the number of samples, which leads to model overfitting in the classification tasks. The FS procedure is applied to reduce the dimensionality of the data while preserving significant information. Considering that the number of features should be equal to or smaller than the sample size (Hua et al., 2005), thus, the following options were chosen: First, the number of selected features was equal to the number of samples in the dataset, while the second option, the number of selected features was 80% of the number of samples. Several methods in the FS procedure (Fig. 1C) were chosen including PCA and the SelectKBest method with ANOVA F-value as a score function. Moreover, ML methods such as LR and RF were chosen to extract important features from trained models. The methods of important feature extraction are different for LR and RF. In the LR model, absolute weights for each feature were ranged in descending order and the top N of them (N is chosen according to the options discussed above) were selected for the next step. RF is a method that fits several decision tree classifiers on various splits of the dataset. The importance of each feature was calculated in several steps: First, the value of the decrease in impurity criterion for each node associated with the feature in each decision tree is calculated, then the sum of calculated values of decrease in impurity criterion on all nodes is multiplied by the percentage of examples reaching these nodes. This weighted sum is a feature of importance in the RF model.

### 2.6 Machine learning methods

Several ML models were trained as shown in Fig. 1C to distinguish between MDD patients and HCs based on selected features only. Different FS methods and parameter combinations inside the ML pipeline selection block were applied including the GridSearchCV procedure (sklearn Python library, version 0.24.2) for fine-tuning the models. For estimation of the model's performance, 10-fold cross-validation with F1 metric was used due to class imbalance. F1-measure on cross-validation for medical datasets was calculated as previously described (Forman and Scholz, 2010). The total number of true positives and false positives over the folds was estimated and then F-measure was computed on them. Similarly, the ROC-AUC and F1 scores were computed. The best-performing models optimized in the pipeline were compared with the baseline model. LR with default parameters and l2 regularization was selected as a baseline. Finally, the top 20 important features were extracted from each best-performing model (on each batch) and each baseline model (for batch1 and batch2) on 10 repeated 10-fold cross-validation, forming a list of top potential biomarkers between MDD patients and HCs groups.

### 2.7 Evaluation measures of the classification performance

In order to provide a performance assessment of the best-built classifiers that were trained on the merged (batch 1 and batch 2) discovery dataset, evaluation measures of the classification performance were performed in independent external transcriptomics datasets. Four well-characterized independent datasets from different populations were retrieved from the Gene Expression Omnibus database (GEO). MDD diagnosis was performed per the DSM-IV criteria (Guze, 1995). The total cohort of 42 MDD patients and 57 HCs among the four external datasets are detailed in Table 1. First, we evaluated the best-built classifiers, which consist of the LR best model along with LR as the feature selection method, in the four confirmation datasets. Next, the best-built classifiers were compared with the baseline classifiers' performance to calculate the relevant performance metrics of our model built compared to the baseline F1-macro in each dataset. We have built a dummy classifier that predicts the most frequent class label. The F1-macro score for the dummy classifier was calculated using the following formula: r/(r+1), where r is the probability of the most frequent class. Since the confirmation datasets showed different gene sets compared to the discovery dataset, subsequently, the best classifiers were trained again using the discovery dataset after removing the genes that were absent in the confirmation dataset. Moreover, to improve the quality of the classification, transfer learning was applied in each external dataset by splitting the dataset into two parts; 80% from the new training dataset and 20% from the new test dataset.

**Table 1:** List of Transcriptomic datasets used in this study.

S. No	Dataset ID	Type of Psychiatric Disorder	Population	Total No. of patients (Visits/Follow-ups)	Comparison of samples		Questionnaires for disease diagnosis and evaluation	Reason for multiple visits
					Non-MDD Patients	MDD patients		
1	GSE99725	MDD	French/Caucasian	33 (24)	18 (13)	15 (11)	DSM4	Follow-up after Bariatric Surgery
							MADRS	
2	GSE76826	MDD	Japanese	32	12 (12)	10 (10)	DSM4	N/A
							SIGH-D	
3	GSE38206	MDD	French/Caucasian	18(18)	9 (9)	9 (9)	DSM4	Follow up in remission state after 8 weeks of MDE
							HDRS	
4	GSE32280	MDD	Chinese	16(16)	8 (8)	8 (8)	DSM4	After treatment with Venlafaxine
							HRSD-17	
5	GSE98793	MDD	German/Caucasian	192	64	128	DSM4 or ICD10	N/A

### 2.8 Functional mapping and annotation of biomarkers to human brain regions

To evaluate the brain structure alterations in MDD patients associated with the identified biomarkers, functional mapping and annotation of the Human brain regions were performed using the Allen Human Brain Atlas (AHBA). The latter is a publicly available resource of comprehensive gene expression mapping of the human brain computing across 3700 spatially different tissues that are collected from six neurotypical postmortem adult brains (Shen et al., 2012).

### 2.9 Occurrence of the identified biomarkers in a large cohort of MDD patients from diverse ethnicities

Cross-validation was performed for the potential DEGs between the MDD patients and HCs that were identified with bioinformatics analysis and ML models from the discovery dataset. Independent cross-validation sets were considered on four well-characterized independent datasets (GSE99725, GSE76826, GSE38206, and GSE32280) from different populations with diverse ethnicities (Caucasian, Japanese and Chinese) (Table 1). First, the *Limma* package under the R software (Ritchie et al., 2015) was used to evaluate the DEGs between MDD patients and HCs in each external dataset at a significance threshold of *p* < 0.05. Next, all described datasets including the discovery and the confirmation external datasets were combined and only the common genes were considered. ML was re-performed on 80% of the common genes among the entire datasets while the performance was evaluated in the 20% holdout.

### 2.10 Recruitment of MDD patients from the Kazakhstan population for biomarker assessment

The identified potential MDD biomarker was further assessed in an independent cohort with a particular genetic background from the Kazakhstan population. Saliva samples of 12 well-characterized MDD patients and 8 HCs were collected at Al-Farabi Kazakh National University, Kazakhstan. The study was approved by the Ethics Committee of the Al-Farabi Kazakh National University (IRB-A083 and IRB-A267). Inclusion criteria were diagnosed with MDD by a psychiatrist for the first time (based on ICD-10) and no medication/treatment. Exclusion criteria were volunteers with substance or alcohol abuse and taking antipsychotic medications/treatment. All participants after signing the consent form completed the Inventory of Depressive Symptomatology (IDS) (Rush et al., 1996). Subjects who showed IDS scores>20 were interviewed by a psychiatrist for diagnosis. Saliva samples were collected from all samples.

### 2.11 mRNA gene expression evaluation using qRT-PCR

The top candidate biomarker *NRG1* was selected for further quantitative gene expression evaluation between MDD patients and HCs from Kazakh population. Saliva samples were stored in RNAlater stabilization solution at +4 °C until analysis. Total RNA was extracted using the RNeasy Mini kit (Qiagen, Germany), according to the manufacturer's protocol. RNA purity and quantity were assessed using Nanodrop2000 (ThermoFisher Scientific, USA). Total RNA was transcribed using a High Capacity cDNA Reverse Transcription Kit (Applied Biosystems, USA) according to the manufacturer's instructions. The primers for *NRG1* gene expression evaluation were Forward:5′-TCGTGGAATCAAACGCTACA-3′ and Reverse:5′-ACTCCCCTCCATTCACACA-3'. 18S rRNA was used as a housekeeping gene with the primers Forward: 5′-TCGCTCCACCAACTAAGAAC-3′ and Reverse: 5′-TGACTCAACACGGGAAAC-3'. The mRNA expression of the candidate and reference genes were quantified in MDD patients and HCs using Maxima SYBR Green/ROX qPCRMaster Mix (ThermoFisher Scientific) on the QuantStudio3 system (Applied Biosystems). The amplification for each sample was carried out in two biological replicates and two technical replicates, then the mean Cq value (quantitation cycle) was used to determine the fold expression change in MDD patients compared to the HCs. The relative expression levels of the target gene were calculated using the 2^−ΔΔCt^ method.

## 3 Results

### 3.1 Identification of the differentially expressed genes among MDD patients

The differential transcriptomic profile across MDD patients and HCs were analyzed using a bioinformatics pipeline, and the result of data distribution before and after quality control, normalization and filtration of the GSE98793 dataset are shown in Fig. 1A. Comparing MDD patients to HCs, a total of 3294 and 2088 differentially expressed genes (DEGs) were identified, respectively, in batch 1 and batch 2 subgroups. The total of DEGs was merged into a common expression matrix based on the gene names to include 1446 common genes between the 106 MDD patients and 51 HCs.

### 3.2 Enrichment analyses of the DEGs in MDD patients

To explore the molecular signatures and biological functions and processes of the identified DEGs in MDD patients compared to the HCs, GSEA was performed. It consists of a computational method that identifies whether a priori set of genes shows statistically significant and coherent differences between two groups across several thousand pre-defined datasets including complex molecular mechanisms, immunologic signatures, biological processes, molecular activities and cellular structures. The enrichment analysis showed that a total of 3262 and 2186 significant pathways (*p* < 0.05) were identified in batch 1 and batch 2, respectively. The GSEA revealed that the DEGs in MDD patients are mainly enriched in pathways related to immune response, immune effector processes, signaling by cytokines, inflammatory response, proinflammatory and profibrotic mediators, cellular responses to stimuli, neurodegeneration pathways, cerebellar atrophy pathway, and neuroactive ligand-receptor interactions. The details of all significant pathways are listed in Supplementary Table 2.

### 3.3 The most informative MDD-related genes from the activated cellular pathways

**Fig. 2:**
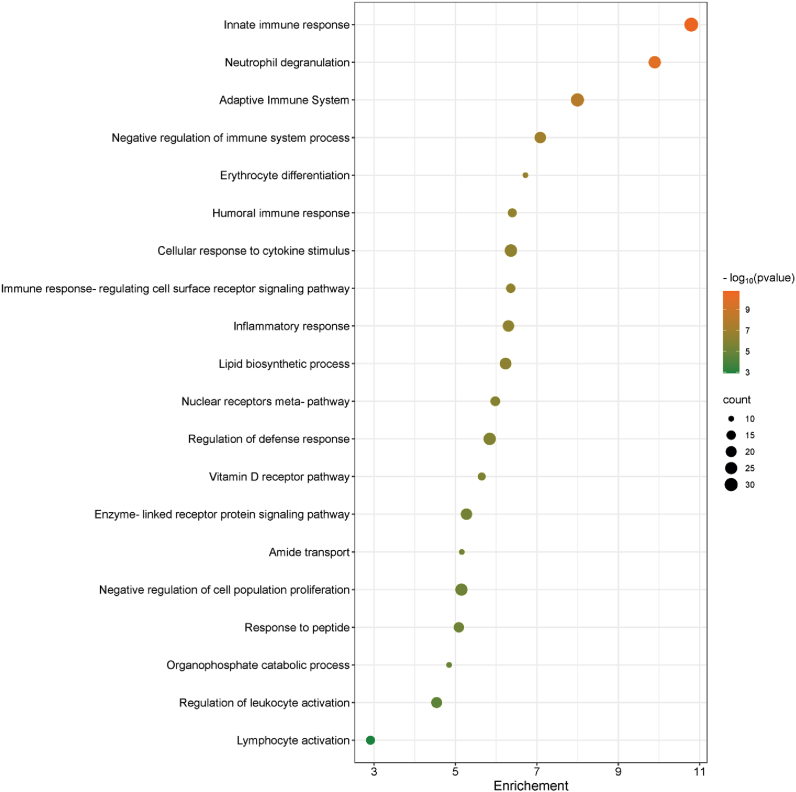
The top 20 enriched pathways in MDD patients compared to healthy controls.

To identify the core genes that potentially determine the significant enrichment of the corresponding identified gene sets, the leading-edge analysis was also performed for all identified significant gene sets in each subgroup of MDD and HCs. Subsequently, the gene frequency was calculated based on the occurrence of a gene amongst all enriched leading-edge core genes from the significant over-represented gene sets in each batch. Overall, 353 frequent genes resulted based on the merged data of MDD patients compared to HCs (Supplementary Table 3). These identified genes could be considered the most informative genes that play or control potential biological roles among the over-enriched pathways in MDD compared to HCs. The functional annotation of these top leading genes in MDD patients further confirms that the immune response-related pathways, the inflammatory responses, and metabolic pathways are the main significantly enriched in MDD compared to HCs (Fig. 2). Next, only genes with large gene expression FC > 1.5 or <0.5 between MDD patients and HCs were considered. Hence, 28 significantly important genes between MDD patients and HCs were left as shown in Table 2.

**Table 2:** Filtered Top leading genes in MDD patients compared to healthy controls.

NAME	Gene Description	Fold Change
*AHSP*	alpha hemoglobin stabilizing protein	1.64
*ANKRD22*	ankyrin repeat domain 22	1.76
*BPGM*	bisphosphoglycerate mutase	1.55
*BPI*	bactericidal permeability increasing protein	1.85
*CACNA1E*	calcium voltage-gated channel subunit alpha1 E	1.77
*CEACAM8*	CEA cell adhesion molecule 8	2.50
*CLEC12B*	C-type lectin domain family 12 member B	1.82
*CMPK2*	cytidine/uridine monophosphate kinase 2	1.62
*DEFA4*	defensin alpha 4	2.85
*DSC2*	desmocollin 2	1.51
*FOLR3*	folate receptor gamma	1.54
*HP*	haptoglobin	2.06
*IFI44L*	interferon induced protein 44 like	1.52
*IFI6*	interferon alpha inducible protein 6	1.56
*IFIT1*	interferon induced protein with tetratricopeptide repeats 1	1.58
*ITGA2B*	integrin subunit alpha 2b	1.61
*LCN2*	lipocalin 2	2.32
*LTF*	lactotransferrin	2.85
*NRG1*	neuregulin 1	1.68
*OLFM4*	olfactomedin 4	3.57
*ORM1*	orosomucoid 1	1.69
*RSAD2*	radical S-adenosyl methionine domain containing 2	1.71
*SERPING1*	serpin family G member 1	1.51
*SOX6*	SRY-box transcription factor 6	1.51
*TCN1*	transcobalamin 1	1.67
*TDRD9*	tudor domain containing 9	1.70
*THBS1*	thrombospondin 1	1.61
*XK*	X-linked Kx blood group antigen, Kell and VPS13A binding protein	1.57

### 3.4 Explainable machine learning models

To identify the most sensitive and potent predictive MDD-related genes, we further evaluated the above-resulted DEGs between MDD and HCs with another computational method by applying different ML models. The fine-tuned parameters for the best-built models in each subgroup comparing MDD patients to HCs were presented in Table 3. Among all tested models, the best results were obtained with 3 final models based on the best-performing ML algorithms on separate and merged batches Table 4. The best model obtained on the merged dataset is LR with LR as the FS method (number of selected features = 157). The final model's score is 0.76 ± 0.11 and ROC-AUC is 0.82 ± 0.12. While, the best model in batch1 is LR (F1 = 0.73 ± 0.17, ROC-AUC = 0.88 ± 0.13) and in batch2 is SVM (F1 = 0.7 ± 0.16, ROC-AUC = 0.79 ± 0.15). Additionally, the performance classification of the best-built classifiers trained on the merged discovery dataset which consists of the LR best model along with LR as the feature selection method was further evaluated in four well-characterized independent transcriptomics datasets from different populations. To compare the performance of the best model with baseline F1-macro, we have built a dummy classifier that predicts the most frequent class label. Due to batch effect and domain shift, the F1-macro score was similar to the baseline classifier in the majority of the datasets (Table 5). The batch effect appears due to differences in equipment settings and experiment design which leads to changes in the features' distribution between training data and external confirmation datasets and thus results in poor classification performance. Hence, to resolve that, transfer learning was additionally applied to show that the classification performance in 10-fold cross-validation has improved for all datasets compared to the classifiers without pretraining, and in all cases, the F1-macro score was better than the baseline as shown in Table 5.

**Table 3:** Final ML model parameters.

	GSE98793: Batch 1	GSE98793: Batch 2	GSE98793: Batch 1 + Batch 2
Model	**LR (baseline)**	**LR (baseline)**	**-**
Parameters	C = 1, penalty = 'l1', solver = 'liblinear'	C = 1, penalty = 'l2', solver = 'liblinear'	**-**
Model	**VarianceThreshold + LR**	**VarianceThreshold + SVM**	**LR (157) + LR**
Parameters	C = 1, class_weight = balanced, penalty = l1, solver = liblinear	C = 0.1, class_weight = balanced, kernel = linear, probability = True	LR (157): default parameters C = 3, class_weight = balanced, solver = liblinear
Model	**RF (80)+XGB**	**RF (77)+XGB**	**LR (136)+SVM**
Parameters	RF (80): n_estimators = 8 learning_rate = 0.3, max_depth = 6, eta = 0.1, remaining parameters -default	RF (77): n_estimators = 8 learning_rate = 0.3, max_depth = 6, eta = 0.3, remaining parameters -default	LR (136): default parameters C = 0.01 class_weight = 'balanced', kernel = 'linear'
Model	**KBest (80)+SVM**	**KBest (77)+LR**	**KBest (157)+RF**
Parameters	SelectKBest (80): default parameters class_weight = 'balanced', kernel = 'linear'	SelectKBest (77): default parameters C = 0.1, class_weight = 'balanced', penalty = 'l1′,	SelectKBest (157): default parameters class_weight = 'balanced', max_depth = 7, n_estimators = 10,

In the pipeline final model is consist of a feature selection method (optional) and a predictive model. If a feature selection method is used it is specified with the number of selected features in the brackets, otherwise, only the predictive model is specified (trained on all features). LR: Logistic Regression, SVM: Support Vector Machine Classifier, XGB: XGBoost.

**Table 4:** Final model's performance.

Feature selection + Model	Batch 1				Batch 2				Batch 1 + Batch 2		
	Baseline	LR	RF (80)+XGB	KBest (80)+SVM	Baseline	SVM	KBest (77)+LR	RF (77)+XGB	LR (157)+LR	LR (136)+SVM	KBest (157)+RF
Accuracy:	0.7 ± 0.15	0.79 ± 0.11	0.71 ± 0.11	0.74 ± 0.17	0.72 ± 14	0.75 ± 0.13	0.71 ± 0.17	0.71 ± 0.12	0.8 ± 0.1	0.78 ± 0.10	0.69 ± 0.09
ROC AUC	0.78 ± 0.18	0.88 ± 0.13	0.72 ± 0.2	0.7 ± 0.24	0.72 ± 0.18	0.79 ± 0.15	0.73 ± 0.18	0.68 ± 0.24	0.82 ± 0.12	0.82 ± 0.13	0.7 ± 0.14
F1	0.63 ± 0.18	0.73 ± 0.17	0.63 ± 0.17	0.68 ± 0.22	0.64 ± 0.18	0.7 ± 0.16	0.68 ± 0.18	0.64 ± 0.17	0.76 ± 0.11	0.74 ± 0.12	0.63 ± 0.11

MDD vs HC classification task was based on transcriptomic data from two batches separately and from their combination. Here, feature selection methods and ML models are specified together with the number of selected features in brackets. RF - Random Forest, XGB -XGBoost Classifier, LR -Logistic Regression, SVM -Support Vector Machine Classifier, LR (157) - Logistic Regression for feature selection, where 157 features were selected**.** If the number of features is not specified in the brackets, the model is trained on all features.

**Table 5:** The classification performance comparison (F1-macro) with and without retraining compared to a baseline classifier in external transcriptomics datasets.

External Dataset	Baseline classifier (dummy)	Best classifier without retraining	Best classifier with retraining
GSE76826 (22 samples)	0.34 ± 0.02	0.6	0.76 ± 0.2
GSE38206 (18 samples)	0.37 ± 0.03	0.35	0.66 ± 0.22
GSE99725 (33 samples)	0.35 ± 0.01	0.32	0.48 ± 0.19
GSE32280 (16 samples)	0.4 ± 0.01	0.35	0.47 ± 0.34

### 3.5 Relevant and stable MDD features identified from the best-built ML models

For the discovery dataset, the performances of all models showed ≥75% of accuracy for both joint and separate batches, consequently, we decided to include features from the best models of the separate and merged data for the downstream analysis. From the best 3 identified models, the most informative and stable features were extracted as MDD-candidates biomarkers including the top 20 important features for batch 1, batch 2 and merged batches. We also included the top 20 important features from baseline models for batch 1 and batch 2. The score for each feature was computed as a share of all folds, where this particular feature was selected by the model during the FS step. In this experiment, we performed a total of 100 folds of cross-validation with 10 runs of a 10-fold cross-validation procedure for each group of data to estimate the reliability of the obtained features. Next, after selecting the important features, post-hoc analysis (scipy Python library, version 1.9.1) was carried out to evaluate the significant differences in the gene expression between MDD patients and HCs. To assess the applicability of the T-test, the Shapiro test was applied to evaluate the normality distribution and Levene's test was used to test the equality of variances, but if these assumptions were not met, the Mann-Whitney *U* test was applied. For each comparison of the separate and merged datasets of MDD patients compared to HCs, the significant important features are presented, respectively, in Supplementary Tables 4–6.

### 3.6 MDD-related biomarkers emerged from data integration

**Fig. 3:**
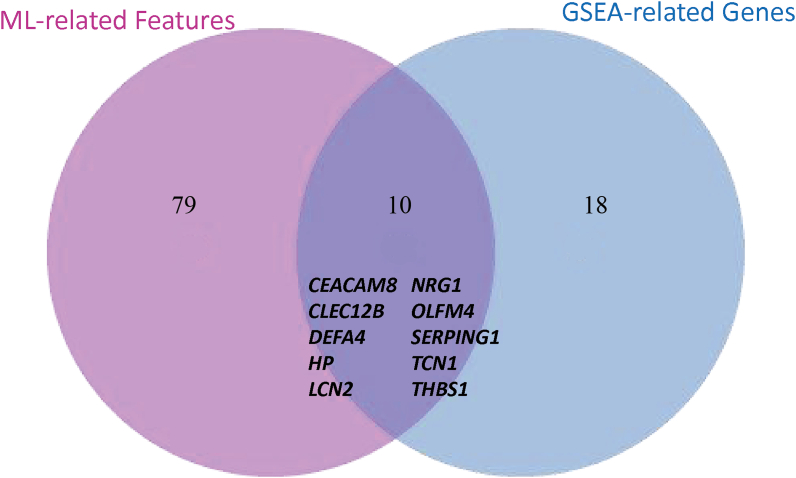
Venn diagram showing the overlap between the significantly identified genes by machine learning models and GSEA-leading gene analysis in MDD patients compared to healthy controls.

The identified gene lists from ML final models and the top frequent genes from the GSEA on the merged data from batch 1 and batch 2 results were intersected to identify the MDD-related biomarkers. Since ML models are based on different principles and therefore may reveal different biomarkers, thus, the important features from the best three models were extracted to increase the set of overlapped biomarkers from the two approaches. The overlapping between the 86 most important features resulting from ML models and the 28 top leading genes resulting from the GSEA showed 10 common genes (Fig. 3) including *CEACAM8, CLEC12B, DEFA4, HP, LCN2, NRG1, OLFM4, SERPING1, TCN1* and *THBS1*.

### 3.7 Mapping of the MDD-related biomarkers across the human brain regions

**Fig. 4:**
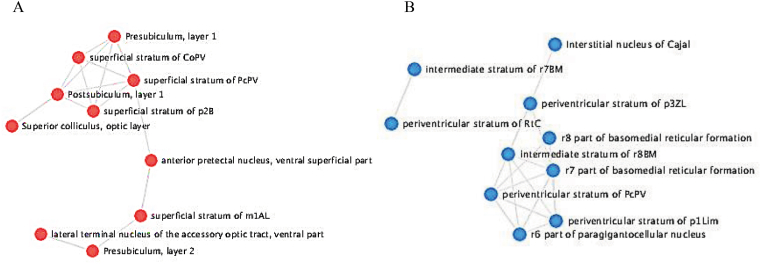
Network intersections of brain regions involving A) up-regulated and B) down-regulated genes profiled by the Allen Brain Atlas.

To get a better understanding of the association between brain structural alterations and gene expression changes in MDD, we assessed the brain-wide gene expression changes of the identified MDD-related biomarkers, using the whole-brain transcriptomic dataset from the AHBA. The mapping of the 10 MDD-related biomarkers showed significant (*p* < 0.05) related up-and down-regulated genes across the brain regions as listed in Table 6. The network analysis showed close interconnections between the different involved brain regions suggesting that the evaluated genes are biologically interconnected as a group in different cognitive functions and varied range of behaviors (Fig. 4). Thus, the top identified MDD-related biomarkers are capable of modeling multivariate associations between brain plasticity and behavior disorders.

**Table 6:** Significantly enriched brain regions profiled by Allen Human Brain Atlas of the MDD-related genes.

Brain region	P-value	MDD-related Genes	Status in the brain region
Periventricular stratum of PcPV	0.0093	*HP;THBS1*	down-regulated
Periventricular stratum of RtC	0.0093	*CLEC12B;OLFM4*	down-regulated
Intermediate stratum of r7BM	0.0093	*HP;THBS1*	down-regulated
Intermediate stratum of r8BM	0.0093	*LCN2;THBS1*	down-regulated
Periventricular stratum of p1Lim	0.0093	*CLEC12B;HP*	down-regulated
Periventricular stratum of p3ZL	0.0093	*CLEC12B;OLFM4*	down-regulated
Interstitial nucleus of Cajal	0.0093	*HP;THBS1*	down-regulated
r6 part of paragigantocellular nucleus	0.0093	*HP;THBS1*	down-regulated
r7 part of basomedial reticular formation	0.0093	*LCN2;THBS1*	down-regulated
r8 part of basomedial reticular formation	0.0093	*LCN2;THBS1*	down-regulated
r8 part of gigantocellular reticular area	0.0093	*LCN2;THBS1*	down-regulated
Retrohypothalamic nucleus	0.0093	*CLEC12B;OLFM4*	down-regulated
Mantle zone of r7BI	0.0093	*HP;LCN2*	down-regulated
Nucleus of reunions	0.0093	*CLEC12B;OLFM4*	down-regulated
Mantle zone of r7BM	0.0093	*HP;THBS1*	down-regulated
Mantle zone of r8BM	0.0093	*LCN2;THBS1*	down-regulated
Shell of p3ZL	0.0093	*CLEC12B;OLFM4*	down-regulated
Intermediate part of r7B	0.0093	*HP;LCN2*	down-regulated
Medial part of r7B	0.0093	*HP;THBS1*	down-regulated
Medial part of r8B	0.0093	*LCN2;THBS1*	down-regulated
p1Lim part of periaqueductal gray	0.0093	*CLEC12B;HP*	down-regulated
Paraventricular hypothalamic nucleus, descending division	0.0093	*CLEC12B;OLFM4*	down-regulated
Paraventricular hypothalamic nucleus, descending division, lateral parvicellular part	0.0093	*CLEC12B;OLFM4*	down-regulated
Paraventricular hypothalamic nucleus, magnocellular division, posterior magnocellular part	0.0093	*CLEC12B;OLFM4*	down-regulated
Paraventricular hypothalamic nucleus, magnocellular division, posterior magnocellular part, lateral zone	0.0093	*CLEC12B;OLFM4*	down-regulated
Paraventricular nucleus, cap part	0.0093	*CLEC12B;OLFM4*	down-regulated
Lateral terminal nucleus of the accessory optic tract, ventral part	0.0004	*HP;SERPING1;OLFM4*	up-regulated
Superior colliculus, optic layer	0.0004	*HP;LCN2;THBS1*	up-regulated
Anterior pretectal nucleus, ventral superficial part	0.0004	*HP;SERPING1;OLFM4*	up-regulated
Superficial stratum of CoPV	0.0004	*HP;SERPING1;OLFM4*	up-regulated
Superficial stratum of PcPV	0.0004	*HP;SERPING1;OLFM4*	up-regulated
Superficial stratum of m1AL	0.0094	*HP;LCN2*	up-regulated
superficial stratum of p2B	0.0094	*SERPING1;THBS1*	up-regulated
Postsubiculum, layer 1	0.0094	*HP;LCN2*	up-regulated
Presubiculum, layer 1	0.0094	*SERPING1;OLFM4*	up-regulated
Presubiculum, layer 2	0.0094	*SERPING1;OLFM4*	up-regulated
Lateral part of Med	0.0094	*OLFM4;THBS1*	up-regulated
Layer 2 of PrS	0.0094	*SERPING1;OLFM4*	up-regulated
m1Lim part of the midbrain reticular formation	0.0094	*HP;LCN2*	up-regulated
Medial amygdalar nucleus, posteroventral part	0.0094	*LCN2;SERPING1*	up-regulated
Medial (fastigial) cerebellar nucleus	0.0094	*OLFM4;THBS1*	up-regulated
Subbrachial nucleus, caudal part	0.0094	*HP;LCN2*	up-regulated
Nucleus of the inferior collicular brachium, rostral part	0.0094	*HP;LCN2*	up-regulated
Superficial layers of TG	0.0094	*LCN2;THBS1*	up-regulated
Intermediate stratum of m1AL	0.0094	*HP;LCN2*	up-regulated
Intermediate stratum of m1Lim	0.0094	*HP;LCN2*	up-regulated
Presubiculum	0.0253	*SERPING1;OLFM4*	up-regulated

### 3.8 Reproducibility of MDD-related biomarkers expression changes across different populations

To evaluate the identified MDD-related biomarkers in another cohort of 42 MDD patients and 57 HCs, independent external transcriptomic datasets were selected including different populations with diverse ethnicities (Caucasian, Japanese and Chinese). Transcriptomic analysis was performed for each dataset separately, then identification of DEGs was proceeded for each comparison of MDD patients compared to HCs and also for the follow-up of patients e.g. before/after surgery, remission and treatment. All studied comparisons were summarized in Table 7. The 10 identified MDD-related biomarkers were evaluated across different comparisons to show that all these genes, except for the *OLFM4* gene, were showing gene expression changes in at least one subgroup of MDD patients (Table 7). Importantly, *NRG1*, neuregulin 1, was the top frequently altered gene in three out of four different MDD populations. In the GSE99725 dataset, the *NRG1* was up-regulated in HCs compared to MDD patients (FC = 0.07, *p* = 0.04) before and after (FC = 0.11, *p* = 0.03) surgery, however, it didn't show a significant variation (*p* > 0.05) once comparing MDD patients before and after surgery. Likewise, in the GSE76826 dataset, the *NRG1* was down-regulated once comparing HCs to MDD patients before remission and after remission (FC = −0.87, *p* = 0.0007) and (FC = −0.85, *p* = 0.0005), respectively, while, no gene expression changes were detected once comparing MDD patients before and after remission. Furthermore, in the case of Venlafaxine treatment in the GSE32280 dataset, the *NRG1* was down-regulated in HCs compared to MDD patients before and after the treatment (FC = −0.44, *p* = 0.04) and (FC = −1.44, *p* = 0.001), respectively, while it was up-regulated when comparing MDD patients before and after Venlafaxine treatment (FC = 1.5, *p* = 0.001). Furthermore, the ML on 80% of the entire combined datasets including the discovery and external datasets along with performance evaluation in the 20% holdout showed that the best-built classifiers trained on the merged discovery dataset which consists of the LR best model along with LR as the feature selection method showed a 0.58 ± 0.05 F1 macro score on 5-fold cross-validation which is better than baseline with 0.38 ± 0.04 F1 macro score. Importantly, the *NRG1* gene was considered among the most performing genes to distinguish between MDD patients and HC groups among the independent populations with diverse ethnicities. Taking these results together, we suggest *NRG1* as a potential robust biomarker to differentiate between MDD patients and HCs under different depression-related conditions as well as to follow up the MDD patients' remission.

**Table 7:** Cross-Validation of the top MDD-related biomarkers across different populations of MDD patients and healthy controls.

GSE No.	Comparison Groups	No. of Controls	No. of MDD Patients	Overlapping DEGs
GSE99725	Healthy Controls (Pre-Bariatric Surgery) vs MDD Patients (Pre-Bariatric Surgery)	18	15	NRG1 **↑**, SERPING1↑
	Healthy Controls (Post-Bariatric Surgery) vs MDD Patients (Post-Bariatric Surgery)	13	11	NRG1 ↑, CLEC12B ↑, HP↑
	MDD Patients (Pre-Bariatric Surgery) vs MDD Patients (Post-Bariatric Surgery)	15	11	LCN2 ↑, HP↑, CEACAM8↑
GSE76826	Healthy Controls vs MDD Patients	12	10	NRG1 ↓
	Healthy Controls vs MDD Patients in Remission	12	10	NRG1 ↓, HP↑
	MDD Patients in Remission vs MDD Patients	10	10	HP↓
GSE32280	Healthy Controls vs MDD Patients (Pre-Venlafaxine Treatment)	8	8	NRG1 ↓, THBS1 ↑
	Healthy Controls vs MDD Patients (Post-Venlafaxine Treatment)	8	8	NRG1 ↓
	MDD Patients (Pre-Venlafaxine Treatment) vs MDD Patients (Post-Venlafaxine Treatment)	8	8	NRG1 ↑
GSE38206	Healthy Controls vs MDD Patients	9	9	LCN2 ↑, THBS1 ↓, DEFA4 ↓, HP↓, TCN1↑, CEACAM8↑
	Healthy Control vs MDD Patients in Remission	9	9	LCN2 ↑, SERPING1 ↑, THBS1 ↓, TCN1↑, CEACAM8↑
	MDD Patients in Remission vs MDD Patients	9	9	HP↑

### 3.9 NRG1 is a liquid biopsy and brain-related biomarker

**Fig. 5:**
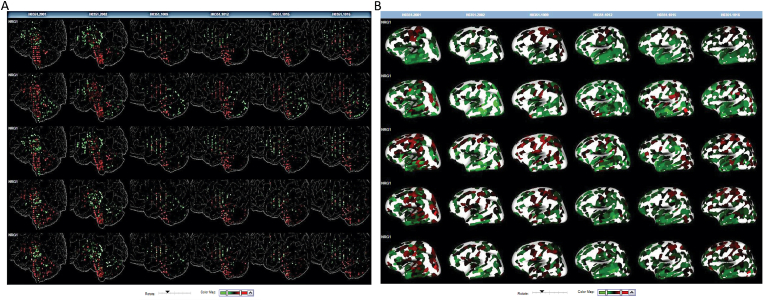
Gene expression proofing of the NRG1 gene in brain samples A) Subcortex, B) Left cortex from healthy samples.

**Fig. 6:**
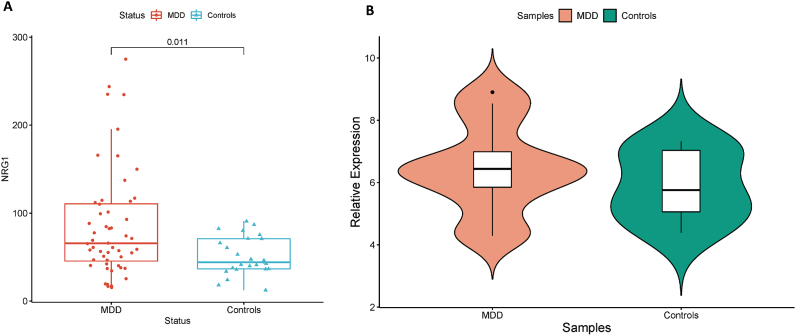
Increased expression of *NRG1* gene in MDD patients compared to Healthy controls. A) *NRG1* was up-regulated in MDD patients compared to Healthy controls from the Microarray dataset. Y-axis is showing a random unit of expression. B) Confirmation of the *NRG1* up-regulation in MDD patients compared to Healthy controls using RT-QPCR in an independent population. Y-axis is showing relative expression.

To evaluate the potential of the *NRG1* gene as an MDD-related diagnosis biomarker, first, we checked its gene expression in the Human brain using the AHBA. The *NRG1* expression was assessed among transcriptomic data of six post-mortem brains from health samples without known neuropsychiatric or neuropathological disorders history. As shown in Fig. 5, the *NRG1* is expressed in the left cortex and subcortex with variable intensity among the six studied samples, suggesting that the *NRG1* is a brain-related biomarker that could be informative upon a mental disorder such as depression. Due to the inaccessibility of the brain tissues, saliva biomarkers are considered the ultimate non-invasive alternative for mental-related diseases. To assess the possibility of using *NRG1* as a potent MDD-related biomarker in saliva samples, we investigated the presence of *NRG1* using quantitative RT-PCR on 12 patients with MDD and 8 HCs from the Kazakh population. The latter has a particular population ethnicity and is different from the other populations that were explored in the discovery and external datasets. The relative quantification of *NRG1* gene expression after normalization to the reference gene 18S rRNA showed an up-regulation by the factor 1.48 in MDD patients compared to HCs (Fig. 6).

## 4 Discussion

**Fig. 7:**
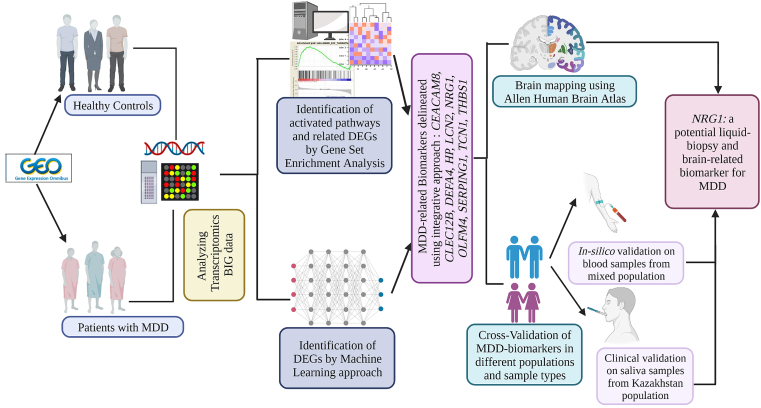
The general workflow of MDD-related Biomarkers identification based on Bioinformatics and ML-approach to connect Transcriptomic changes and Brain regions. The figure was generated by Biorender tool at: https://app.biorender.com/

In the present study, we applied a mixture of bioinformatics and ML approaches to mine transcriptomics data of patients with MDD compared to HCs. The study workflow is summarized in Fig. 7. The integrative analysis of the bioinformatics and ML identified 10 potent MDD-related biomarkers including *CEACAM8, CLEC12B, DEFA4, HP, LCN2, NRG1, OLFM4, SERPING1, TCN1* and *THBS1,* that showed the most significant and strong differential expression between MDD patients and HCs. Importantly, the identification of such biomarkers would provide a better preventative intervention, and insight into patients' responses to anti-depressant therapy, further aiding in monitoring the effectiveness of treatment in patients (Li et al., 2021b). Therefore, the development of specific depression-related biomarkers is crucial to aid in the diagnosis and monitoring of patients with depression which may result in earlier diagnosis, as well as earlier intervention, and substantially improve the patients' outcomes (Nobis et al., 2020).

To identify the most sensitive and potent predictive MDD-related genes, different ML models were applied for the DEGs between MDD and HCs in the discovery dataset (GSE98793). The fine-tuned parameters for the best-built models in each subgroup comparing MDD patients to HCs showed that the best model was obtained on the merged dataset consisting of LR with LR as the FS method (number of selected features = 157) with a final model's score is 0.76 ± 0.11 and ROC-AUC is 0.82 ± 0.12. Furthermore, evaluation of the classification performance measures of this latter best-built classifiers was performed in four independent external transcriptomics datasets. Overall, the confirmatory analysis showed that for each dataset there are differing numbers of gene sets with different batch effects and other type 1 errors inherent within the samples related to the transcriptomics platform and the recruited HC and MDD patients. Although this resulted in a different set of genes for each dataset (including the discovery and the external confirmation datasets), our findings showed that the best-built classifiers with retraining generally performed much better than the best classifier without retraining compared to the baseline classifier, for example for the dataset GSE76826 the F1-score was 0.34 with baseline classifiers, 0.6 with best classifiers without retraining and 0.76 with best classifiers with retraining. Additionally, when we retrained the best-built model on each external dataset the important features remained the same and the values of the LR coefficients didn't change significantly. Thus, by applying transfer learning only the value of coefficients changes, while the prediction quality is improved significantly. The results would seem to demonstrate that the best-built classifiers are better not to be applied as are due to domain adaptation problems such as changes in distributions resulting from different hardware settings and experimental variation, however, using transfer learning and retraining the classifiers on external data can improve the classification performance. These results correlate favorably with Pang and Yang's findings (Pan and Yang, 2010) and further support the relationship between transfer learning and other related machine learning techniques such as domain adaptation, and sample selection bias.

The enrichment analyses of the DEGs in MDD patients are significantly related to the immune response and the inflammatory responses. These findings are consistent with previous transcriptomics studies in MDD which identified important biomarkers focusing on inflammation, neuroplasticity, neurotransmitters and stress-related genes (Mariani et al., 2021). Moreover, this is in line with Wang et al. findings showing that a whole transcriptome analysis in the peripheral blood of MDD patients reported that inflammation and metabolism-related pathways are the main over-represented pathways probably involved in the pathophysiology of MDD (Wang et al., 2022).

Depression is expected to distress the functional activity of the brain. More recent evidence highlights that depression affects mainly three portions of the brain regions including the hippocampus (resides in the temporal lobe of the brain), the pre-frontal cortex (located at the front of the frontal lobe) and the amygdala (located at the frontal portion of the temporal lobe) (Zhang et al., 2018). Importantly, we showed that by mapping the identified MDD-related biomarkers across the Human brain regions, significant overlapped differentially expressed genes across the brain regions have resulted. Furthermore, the mapping network analysis showed close interconnections between the different involved frontal brain regions suggesting that the evaluated genes are significantly associated with cognitive functions which are probably altered in depression. This is in agreement with previous findings showing that patients with MDD are characterized by decreased brain activation in frontal areas and increased activation in limbic regions (Zhong et al., 2016), with the frontal lobe proposed to be the most common region to manifest anatomic abnormalities in MDD compared to the other brain lobes (Srivastava et al., 2016). There is also evidence of hyperactivity in the dorsolateral prefrontal cortex, left inferior frontal gyrus, and rostral anterior cingulate gyrus (Wagner et al., 2006). Notably, we ascertained that out of the ten identified MDD-related biomarkers, six biomarkers (*CLEC12B, HP, LCN2, OLFM4, SERPING1,* and *THBS1*) were related to a specific subset of brain regions, however, *CEACAM8, DEFA4, NRG1, TCN1* do not show a distinct differential expression on the brain areas of healthy cases. These findings appear to be substantiated by previous studies revealing that identified hub-genes in peripheral blood samples are capable to classify MDD from HCs with high accuracy, although some of these reliable markers were not correlated with the brain differential gene expression (Gomez Rueda and Bustillo, 2022). These results point to the probability that due to brain plasticity, not necessarily all mental-related biomarkers follow the usual computational analysis hypothesis. That is why applying the ML and computational approaches is of the highest importance to retrieve hidden key biological indicators.

To further evaluate the reproducibility of identified MDD-related biomarkers expression changes across different populations, we performed cross-validation in another cohort with different conditions of depression including MDD patients and HCs from independent external confirmation transcriptomic datasets. Interestingly, the *NRG1* gene resulted as the most robust biomarker to differentiate between MDD patients' and HCs under different depression-related conditions and as well to follow-up the MDD patients’ remission. Furthermore, we performed a clinical evaluation of the *NRG1* in saliva samples of well-characterized MDD patients and HCs from the Kazakhstan population using RT-qPCR. As we first showed in the discovery transcriptomic dataset, the up-regulation of *NRG1* in MDD patients (*p* = 0.011), the over-expression was further validated in our MDD patients but with no statistical significance (*p* > 0.05), likely due to the small sample size. These findings led us to suggest *NRG1* as a liquid-biopsy biomarker for MDD diagnosis and prognosis.

*NRG1*, coding for neuregulin 1, is a member of the epidermal growth factor (EGF) family of extracellular ligands that present an important role in the maintenance, development and reparation of both the central nervous system (CNS) and peripheral nervous system (PNS) through the ErbB signaling pathway (Mei and Nave, 2014). Several clinical and genetic association studies have reported the linkage between *NRG1* gene variations and susceptibility to different psychiatric disorders including depression (Wen et al., 2016; Levchenko et al., 2020; Mitchell et al., 2022). Furthermore, changes in *NRG1* isoform expression levels in the post-mortem pre-frontal cortex of patients with major psychiatric disorders such as schizophrenia and unipolar depression have been consistently reported (Hashimoto et al., 2004; Bertram et al., 2007). A clinical study with 267 MDD patients suggested that *NRG1*-linked white matter abnormalities were associated with clinical symptoms of depression and anxiety (Duan et al., 2021). Moreover, *NRG1* seems to be a predictive biomarker of the response to pharmacological antidepressant treatment as we showed in our analysis of the dataset (GSE32280), that *NRG1* changes the expression profile in the MDD patients compared to HCs before and after Venlafaxine treatment.

It is worthy of note that *NRG1* plays a critical role in mental-related disorders as it is largely dispersed in the frontal cortex, cerebellum and midbrain (Zhang et al., 2017). Importantly, the prefrontal cortex is amongst the critical brain regions that are related to the regulation of the pathological response to stress (Negrón-Oyarzo et al., 2016), fluctuations in behavior, cognitive functions and neuroendocrine responses (Corbett et al., 2019). Furthermore, animal model studies showed that the disturbing *Nrg1* function within the cortical region resulted in decreased synaptic plasticity and augmented inhibitory connections (Clarke et al., 2017). Moreover, mice with heterozygous KO deletion of *Nrg1* showed different schizophrenia-related disorders and advanced sensitivity to stress during adolescence (Samsom and Wong, 2015). Our findings showed an up-regulation of the *NRG1* in the MDD patients compared to HCs in both blood and saliva samples which per several mice models studies showed that increases in the *Nrg1* expression resulted in deficits in the response to a stimulus, hyper-agitation and perturbation in the working memory (Deakin et al., 2009, 2012; Kato et al., 2010; Yin et al., 2013).

Thus, we believe a further investigation of the *NRG1* biomarker in a larger clinical dataset of MDD patients is warranted. The evidence from our study points towards the idea that hybrid AI and Bioinformatics applications can greatly help in the diagnosis and treatment of mental disorders.

## 5 Conclusion

In conclusion, integrative analysis using bioinformatics and ML approaches to compare transcriptomics changes of MDD patients to HCs identified DEGs in MDD patients which are associated with immune response and inflammatory responses pathways. In addition, the analysis identified ten biomarkers panels that can aid in the diagnosis and monitoring of patients with MDD. Amongst those biomarkers, *NRG1* exhibits higher expression in the amygdala and hippocampus brain subregions and was suggested as a non-invasive liquid-biopsy biomarker for the diagnosis of MDD patients.

## Funding

This work was funded by the UoS-Skoltech Collaborative AI for Life award [grant number: AIfoL-2201] to (M.S and R.H), partially supported by the 10.13039/501100006769Russian Science Foundation [grant number: 21-71-10136] to (M.S).

## Data availability

All the relevant data are available in the paper and the Supplementary files. The data used in this study from the sets GSE98793 for the discovery sets and the sets GSE99725, GSE76826, GSE38206 and GSE32280 for confirmation external sets are available from Gene Expression Omnibus (https://www.ncbi.nlm.nih.gov/geo). The machine learning pipeline including sourcecode is uploaded to a publicly available repository at GitHub (https://github.com/maryjis/autoNeuro).

## Declaration of competing interest

None.
