# Supplementary material for: Integrative bioinformatics and artificial intelligence analyses of transcriptomics data identified genes associated with major depressive disorders including NRG1
Source: Neurobiol Stress. 2023 Jul 7;26:100555. doi: 10.1016/j.ynstr.2023.100555 (PMC10423927; doi:10.1016/j.ynstr.2023.100555)
Supplement: Multimedia component 4 [file mmc4.docx]

**Supplementary Table 4: The most informative and stable features (genes) in MDD patients compared to healthy controls (Batch 1).**

| Feature name | Score (%) | p-value | Statistical test |
| --- | --- | --- | --- |
| *LCN2* | 100 | 0.0008 | Mann-Whitney U test |
| *CNTNAP3* | 100 | 0.0053 | Mann-Whitney U test |
| *SERPING1* | 100 | 0.0097 | Mann-Whitney U test |
| *RNF103* | 100 | 0.0011 | T-test |
| *FCER1A* | 90 | 0.0068 | T-test |
| *PDK4* | 90 | 0.0173 | T-test |
| *LINC02273* | 90 | 0.0305 | T-test |
| *ABHD4* | 90 | 0.0262 | T-test |
| *TP53TG3* | 80 | 0.0018 | Mann-Whitney U test |
| *KLRK1* | 80 | 0.0018 | T-test |
| *HOXB-AS1* | 70 | 0.0035 | T-test |
| *PPM1L* | 70 | 0.0174 | T-test |
| *SMPD3* | 60 | 0.0266 | T-test |
| *NME7* | 60 | 0.0010 | Mann-Whitney U test |
| *PYHIN1* | 60 | 0.0013 | T-test |
| *CBS* | 60 | 0.0041 | Mann-Whitney U test |
| *GRB10* | 50 | 0.0002 | T-test |
| *HNRNPM* | 50 | 0.0004 | Mann-Whitney U test |
| *OLFM4* | 50 | 0.0069 | T-test |
| *EIF2B3* | 50 | 0.0055 | T-test |
| *RAB13* | 50 | 0.0002 | T-test |
| *ZNF195* | 50 | 0.0003 | T-test |
| *SCAPER* | 50 | 0.0013 | T-test |
| *CABP7* | 50 | 0.0031 | T-test |
| *DCLRE1B* | 50 | 0.0033 | T-test |
| *NDE1* | 40 | 0.0008 | T-test |
| *R3HDM1* | 40 | 0.0010 | T-test |
| *KAT6A* | 40 | 0.0052 | T-test |
| *CRYZ* | 40 | 0.0071 | Mann-Whitney U test |
| *CEP78* | 30 | 0.0071 | T-test |
| *ARFGEF1* | 30 | 0.0018 | Mann-Whitney U test |
| *NAPRT* | 30 | 0.0038 | Mann-Whitney U test |
| *CCDC50* | 20 | 0.0017 | T-test |
| *KREMEN1* | 20 | 0.0059 | T-test |
| *NIPA1* | 20 | 0.0298 | Mann-Whitney U test |
| *DZIP3* | 20 | 0.0241 | T-test |
| *MGST1* | 20 | 0.0139 | T-test |

**Supplementary Table 5: The most informative and stable features (genes) in MDD patients compared to healthy controls (batch 2).**

| Feature name | Score (%) | p-value | Statistical test |
| --- | --- | --- | --- |
| *SLC26A8* | 100 | 0.0008 | T-test |
| *PROS1* | 100 | 0.0002 | T-test |
| *ERAP2* | 100 | 0.0304 | Mann-Whitney U test |
| *CLEC12B* | 100 | 0.0106 | Mann-Whitney U test |
| *FAM200B* | 90 | 0.0030 | Mann-Whitney U test |
| *CHI3L1* | 90 | 0.0372 | Mann-Whitney U test |
| *NRG1* | 90 | 0.0165 | T-test |
| *APTR* | 90 | 0.0074 | Mann-Whitney U test |
| *CCDC7* | 80 | 0.0052 | T-test |
| *AARS2* | 80 | 0.0073 | Mann-Whitney U test |
| *SRSF6* | 80 | 0.028 | T-test |
| *TTC7B* | 70 | 0.0001 | Mann-Whitney U test |
| *ZP3* | 60 | 0.0081 | Mann-Whitney U test |
| *ITGB5* | 60 | 0.0135 | T-test |
| *PRKCQ-AS1* | 50 | 0.0080 | T-test |
| *HP* | 50 | 0.0023 | T-test |
| *TNFAIP6* | 40 | 0.0021 | T-test |
| *SORT1* | 40 | 0.0108 | T-test |
| *SNX20* | 40 | 0.0074 | Mann-Whitney U test |
| *SLPI* | 40 | 0.0027 | Mann-Whitney U test |
| *OLFM4* | 40 | 0.0098 | T-test |
| *MEF2A* | 40 | 0.0092 | Mann-Whitney U test |
| *MAN1A1* | 40 | 0.0038 | T-test |
| *ITGB3* | 40 | 0.0135 | T-test |
| *EFHC1* | 40 | 0.0009 | T-test |
| *DEFA4* | 40 | 0.0471 | Mann-Whitney U test |
| *CDC42* | 40 | 0.0162 | Mann-Whitney U test |
| *ADAM10* | 40 | 0.0071 | Mann-Whitney U test |
| *CASZ1* | 30 | 0.0191 | Mann-Whitney U test |
| *TRAV5* | 20 | 0.0065 | T-test |
| *SPTBN1* | 20 | 0.0101 | Mann-Whitney U test |
| *F2RL1* | 20 | 0.0142 | Mann-Whitney U test |
| *DOCK4* | 20 | 0.0026 | Mann-Whitney U test |

**Supplementary Table 6: The most informative and stable features (genes) in MDD patients compared to healthy controls (Batch 1 & Batch 2).**

| Feature name | Score (%) | p-value | Statistical test |
| --- | --- | --- | --- |
| *GRB10* | 100 | 0.0045 | Mann-Whitney U test |
| *STAT5B* | 90 | 0.0294 | Mann-Whitney U test |
| *RPS27L* | 80 | 0.0043 | Mann-Whitney U test |
| *SERPING1* | 80 | 0.0196 | T-test |
| *CNTNAP3* | 70 | 0.0007 | Mann-Whitney U test |
| *THEM4* | 70 | 0.0000 | Mann-Whitney U test |
| *OLFM4* | 70 | 0.0001 | Mann-Whitney U test |
| *HP* | 60 | 0.0002 | T-test |
| *B3GNT7* | 50 | 0.0305 | Mann-Whitney U test |
| *NARF* | 50 | 0.0005 | T-test |
| *CAMKK2* | 40 | 0.0090 | T-test |
| *THBS1* | 40 | 0.0466 | Mann-Whitney U test |
| *CLEC12B* | 40 | 0.0113 | Mann-Whitney U test |
| *CEACAM8* | 40 | 0.0002 | Mann-Whitney U test |
| *TMEM33* | 40 | 0.0012 | T-test |
| *ANKRD6* | 40 | 0.0013 | Mann-Whitney U test |
| *TCN1* | 40 | 0.0040 | Mann-Whitney U test |
| *PLEKHA1* | 40 | 0.0019 | T-test |
| *CCDC50* | 40 | 0.0118 | T-test |
| *TTC7B* | 40 | 0.0222 | Mann-Whitney U test |
| *AHI1* | 30 | 0.0142 | Mann-Whitney U test |
| *F5* | 30 | 0.0002 | Mann-Whitney U test |
| *TRAV5* | 30 | 0.0014 | Mann-Whitney U test |
| *RAB13* | 30 | 0.0015 | T-test |
| *ZCCHC2* | 30 | 0.0036 | T-test |
| *PROS1* | 30 | 0.0004 | T-test |
| *FRMD4A* | 30 | 0.0102 | Mann-Whitney U test |
| *C11orf1* | 30 | 0.0157 | Mann-Whitney U test |
